# Gastric biomarkers: a global review

**DOI:** 10.1186/s12957-016-0969-3

**Published:** 2016-08-11

**Authors:** Nick Baniak, Jenna-Lynn Senger, Shahid Ahmed, S. C. Kanthan, Rani Kanthan

**Affiliations:** 1 Department of Pathology and Laboratory Medicine, University of Saskatchewan, 103 Hospital Drive, Saskatoon, SK S7N 0W8 Canada; 2 Department of Surgery, University of Alberta, 116 St & 85 Ave, Edmonton, T6G 2R3, T6G 2B7 AB Canada; 3 Division of Medical Oncology, University of Saskatchewan, 103 Hospital Drive, Saskatoon, SK S7N 0W8 Canada; 4 Department of General Surgery, University of Saskatchewan, 103 Hospital Drive, Saskatoon, SK S7N 0W8 Canada

**Keywords:** Gastric cancer, Biomarkers, Therapy

## Abstract

**Background:**

Gastric cancer is an aggressive disease with a poor 5-year survival and large global burden of disease. The disease is biologically and genetically heterogeneous with a poorly understood carcinogenesis at the molecular level. Despite the many prognostic, predictive, and therapeutic biomarkers investigated to date, gastric cancer continues to be detected at an advanced stage with resultant poor clinical outcomes.

**Main body:**

This is a global review of gastric biomarkers with an emphasis on HER2, E-cadherin, fibroblast growth factor receptor, mammalian target of rapamycin, and hepatocyte growth factor receptor as well as sections on microRNAs, long noncoding RNAs, matrix metalloproteinases, PD-L1, TP53, and microsatellite instability.

**Conclusion:**

A deeper understanding of the pathogenesis and biological features of gastric cancer, including the identification and characterization of diagnostic, prognostic, predictive, and therapeutic biomarkers, hopefully will provide improved clinical outcomes.

## Background

Gastric cancer (GC) has been globally the fourth most commonly diagnosed cancer and the second most lethal malignancy [1, 2]. The data has recently been changing, with WHO GLOBOCAN now reporting GC as the fifth most common cancer and third leading cause of cancer death in both sexes [3]. In 2015, an estimated 24,590 new GC cases and 10,720 GC deaths were diagnosed in the USA [4]. As most patients present with advanced unresectable or metastatic disease at the time of diagnosis, the overall clinical outcome of GC patients remains unsatisfactory, with a 5-year survival rate of less than 30 % [4–6]. The incidence of GC remains high in Japan, but the survival is higher, reported as 52 % [5, 6]. Clinicopathological staging using the TNM system is the major tool used by clinicians to predict GC patient prognosis. However, GC patients of identical TNM stage often exhibit varying clinical outcomes, suggesting that there are additional factors that influence long-term outcomes [7].

GC is a biologically heterogeneous disease that evolves in the background of various genetic and epigenetic alterations. Therefore, it is essential to have a more comprehensive understanding of molecular variables that affect GC disease pathways in order to develop appropriate approaches for its diagnosis and treatment [6]. GC is assumed to originate from a sequential accumulation of molecular and genetic alterations to stomach epithelial cells [8], but the mechanism of carcinogenesis remains complex and poorly understood [9, 10]. Additionally, a number of cellular phenomena, such as tumour microenvironment, inflammation, oxidative stress, and hypoxia, act in parallel with various molecular events to promote initiation, progression, and metastasis of GC [11].

In the traditional Laurén classification, GC is divided into two types: intestinal and diffuse types [12, 13]. The intestinal-type adenocarcinomas characteristically form glands, but with various degrees of differentiation. Intestinal carcinomas are usually diagnosed in older patients, mostly in the antrum, and are strongly attributed to chronic H. pylori infection, with resultant atrophic gastritis, and intestinal metaplasia [12, 13]. Diffuse gastric carcinomas are poorly cohesive, composed of mostly single, or small, nests of neoplastic cells that diffusely infiltrate the gastric wall. This type is found most commonly in the gastric body and in younger patients. Although this type is also associated with H. pylori infection, the carcinogenetic sequence of the diffuse type of GC is not well characterized [13].

**Fig. 1:**
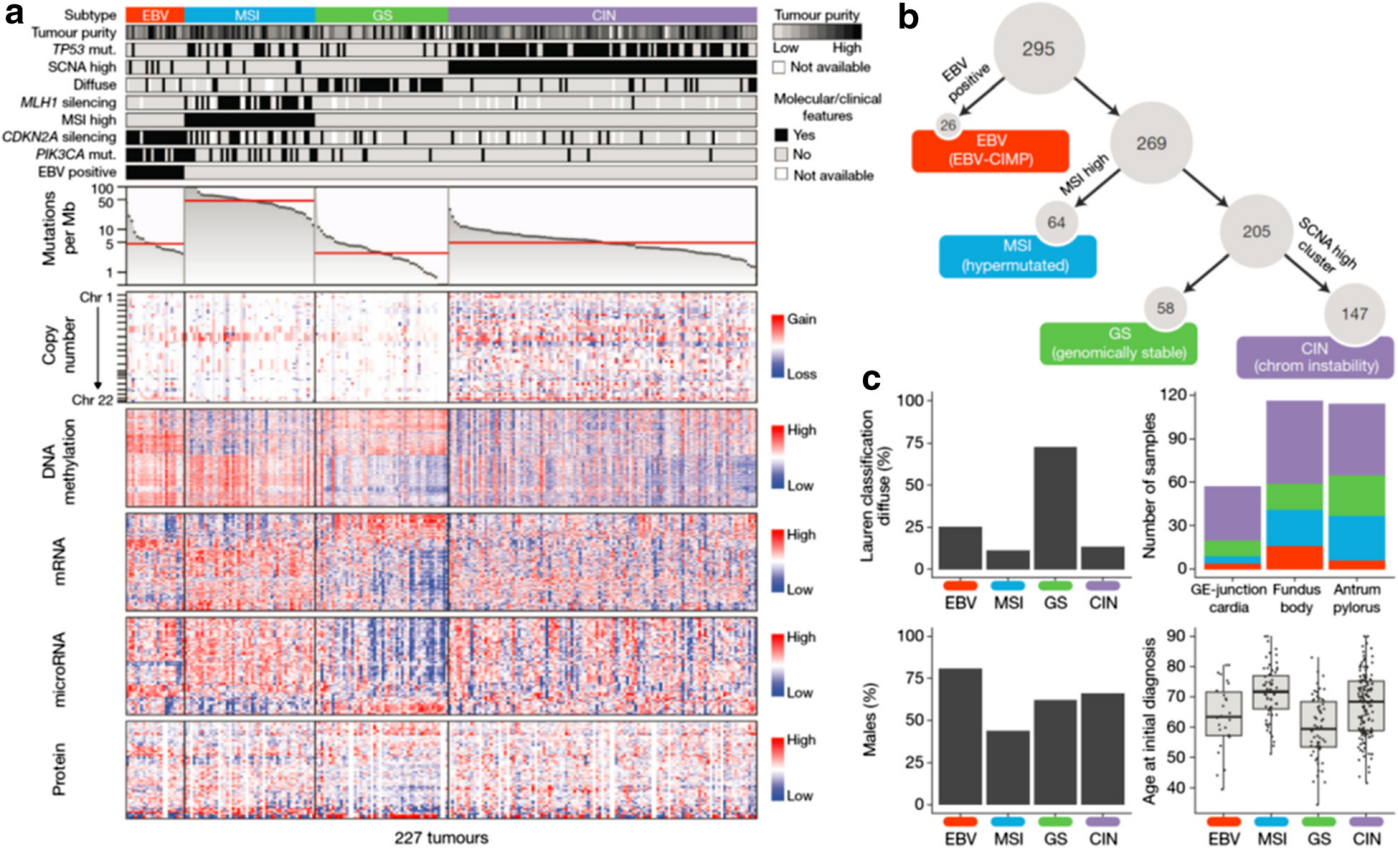
Molecular subtypes of gastric cancer. **a** Gastric cancer cases are divided into subtypes: Epstein–Barr virus (EBV)-positive (*red*), microsatellite instability (MSI, *blue*), genomically stable (GS, *green*), and chromosomal instability (CIN, *light purple*) and ordered by mutation rate. Clinical (*top*) and molecular data (*top and bottom*) from 227 tumours profiled with all six platforms are depicted. **b** A flowchart outlines how tumours were classified into molecular subtypes. **c** Differences in clinical and histological characteristics among subtypes with subtypes coloured as in **a**, **b**. The plot of patient age at initial diagnosis shows the median, 25th and 75th percentile values (*horizontal bar*, *bottom and top bounds* of the box), and the highest and lowest values within 1.5 times the interquartile range (*top and bottom whiskers*, respectively). GE, gastroesophageal (reproduced with permission from The Cancer Genome Atlas Research Network (NATURE | VOL 513 | 11 SEPTEMBER 2014 [14])

**Fig. 2:**
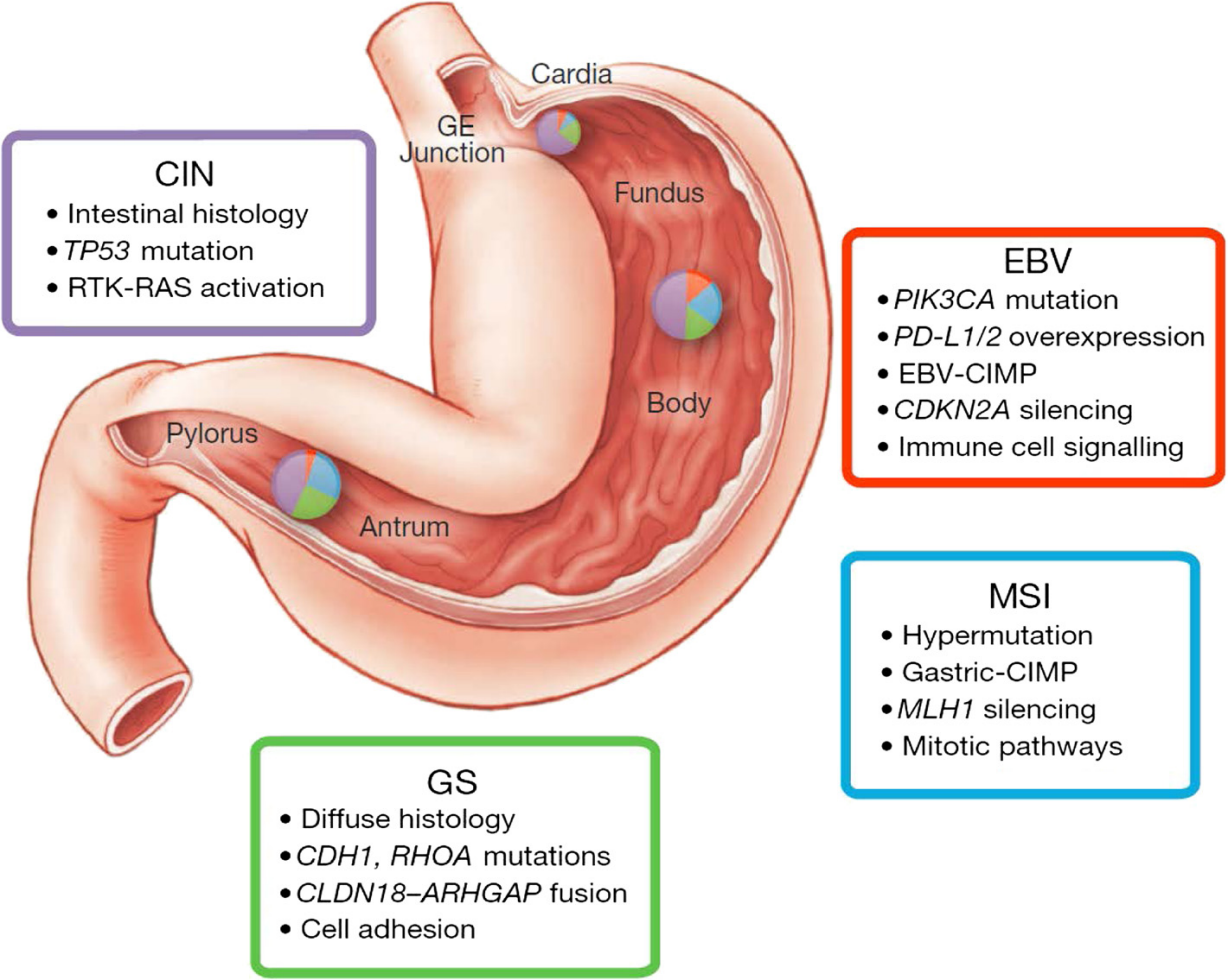
Key features of gastric cancer subtypes. This schematic lists some of the salient features associated with each of the four molecular subtypes of gastric cancer. Distribution of molecular subtypes in tumours obtained from distinct regions of the stomach is represented by inset charts (reproduced with permission from The Cancer Genome Atlas Research Network (NATURE | VOL 513 | 11 SEPTEMBER 2014 [14])

The consortium of The Cancer Genome Atlas (TCGA) has recently reported comprehensive somatic changes in GC and suggested four categories: (i) EBV-positive cases; (ii) microsatellite instability (MSI)-positive cases; (iii) genomically stable (GS) type (near-diploid type); and (iv) chromosomal instability (CIN) type (Table 1, Fig. 1). The EBV-positive tumours have been correlated with PIK3CA mutations; high levels of DNA hypermethylation; and amplification of JAK2, PD-L1, and PDCD1LG2. The MSI tumours display characteristic hypermutation phenotype and downregulation of MLH1 gene. The GS type has been associated with diffuse tumours, mutations of RHOA and CDH1, or fusions involving RHO family GTPase-activating proteins. The CIN tumours have been associated with marked aneuploidy and focal amplification of receptor tyrosine kinases, as well as mutations of TP53 [14] (Fig. 2).

**Table 1:** Features of gastric cancer sub-types defined by TCGA. Based on 295 patients (182 males, 113 females) [14]

Molecular sub-type	Anatomic distribution	Histologic features	Frequency	Molecular
CIN	• 43.0 % antrum • 49.1 % fundus • 64.9 % GEJ/cardia 50.0 % NA	• 26.1 % diffuse • 60.2 % intestinal • 52.6 % mixed • 9.1 % non-specified	• 53.3 % M • 44.2 % F	• TP53 mutation • RTK-RAS activation
EBV	• 5.3 % antrum • 13.8 % fundus • 7.0 % GEJ/cardia	• 7.2 % diffuse • 7.7 % intestinal • 15.8 % mixed • 27.3 % not specified	• 11.5 % M • 4.4 % F	• PIK3CA mutation • PD-L1/2 overexpression • EBV-CIMP • CDKN2A silencing
MSI	• 27.2 % antrum • 21.6 % fundus • 8.8 % GEJ/cardia • 37.5 % NA	• 8.7 % diffuse • 24.5 % intestinal • 15.8 % mixed • 63.6 % not specified	• 15.4 % M • 31.9 % F	• Hypermutation • MLH1 silencing • Gastric CIMP
GS	• 24.6 % antrum • 15.5 % fundus • 19.3 % GEJ/cardia 12.5 % NA	• 58.0 % diffuse • 7.7 % intestinal • 15.8 % mixed	• 19.8 % M • 19.5 % F	• CDH1 mutations • RHOA mutations • CLDN18-ARHGAP fusion

**Fig. 3:**
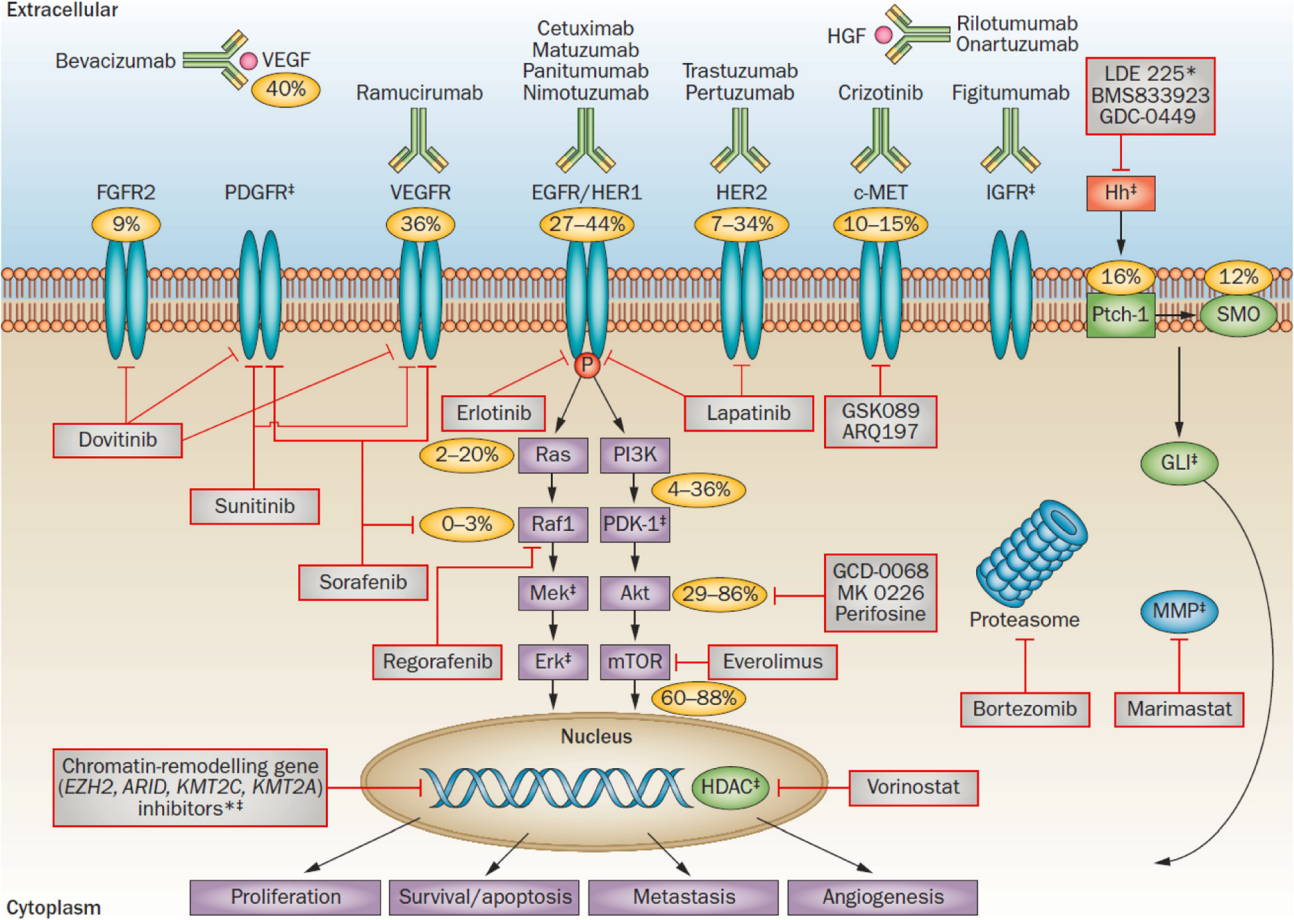
Signalling pathway and targeted therapy in gastric cancer. *Percentages* signify the overall molecular characteristics in the disease: FGFR2 amplification (9 %), VEGF/VEGFR overexpression (36–40 %), EGFR amplification and overexpression (27–44 %), HER2 amplification and overexpression (7–34 %), c-MET amplification (10–15 %), *kRAS* mutation (2–20 %), Raf mutation (0–3 %), PI3K mutation (4–36 %), phospho-Akt expression (29–86 %), phospho-mTOR expression (60–88 %), *PTCH1* overexpression (16%), *SMO* overexpression (12%), and HER3 mutations (10%, not shown). *No clinical trials of these agents have yet been reported in gastric cancer. ^‡^No known numbers or percentages for these genes and pathways. Abbreviations: *EGFR* epidermal growth factor receptor, *FGFR* fibroblast growth factor receptor, *GLI* glioma-associated oncogene family zinc finger 1, *HDAC* histone deacetylase, *HER* human epidermal growth factor receptor, *HGF* hepatocyte growth factor, *Hh* Hedgehog, *IGFR* insulin-like growth factor receptor, *MMP* matrix metalloproteinase, *mTOR* mammalian target of rapamycin, *PDGFR* platelet-derived growth factor receptor, *Ptch-1* protein patched homolog 1, *Smo* smoothened, *VEGF* vascular endothelial growth factor, *VEGFR* vascular endothelial growth factor receptor (reproduced with permission from Wadhwa, R. et al. Nat. Rev. Clin. Oncol. 10, 643–655 (2013) [181])

A deeper understanding of the pathogenesis and biological features of GC is necessary to further inform and enhance early detection and treatment methods. The discovery of new biomarkers and their application, in conjunction with traditional cancer diagnosis, staging, and prognosis, will help to improve early diagnosis and patient care. The search for cancer biomarkers is carried out in order to identify tumour cells at early stages and predict treatment response, ultimately leading to a favourable therapeutic outcome [15]. Biomarkers are predominantly of four types, diagnostic, predictive, prognostic, and therapeutic. A diagnostic biomarker is a noninvasive marker for the detection of early disease. A prognostic biomarker provides information on the likely course of disease and thus yields important information about therapy outcomes and patient survival as well as provide suggestions for further treatment [15, 16]. In contrast, a predictive biomarker is defined as a marker that can be used to identify subpopulations of patients who are most likely to respond (or not) to a targeted therapy [17]. An ideal predictive marker should be reliable, readily available, and detectable by reasonably acceptable laboratory techniques [18]. A therapeutic biomarker is a potential target for cancer therapy (Fig. 3). Therapeutic targets are usually target proteins that are identified as potential biomarkers for cancer but lack accurate clinical evidences or trials to evaluate their position within the history of cancer progression [16].

In this manuscript, a global review on the gastric biomarker literature to date is undertaken, which is dedicated exclusively to the discussion of the role of biomarkers in GC, specifically HER2; E-cadherin; fibroblast growth factor receptor (FGFR)/human epidermal growth factor receptor family (EGFR)/mammalian target of rapamycin (mTOR)/hepatocyte growth factor receptor (HGFR, MET); PD-L1 expression; TP53; MSI; and emerging biomarkers including microRNAs, long noncoding RNAs (LncRNAs), and matrix metalloproteinases (MMPs) (Table 2). An English literature search on MEDLINE combining the terms “gastric cancer” and “biomarkers” retrieved 801 manuscripts between the years of 1995 and 2015. The primary manuscripts and their relevant secondary references were reviewed.

**Table 2:** Frequency of co-mutations in gastric cancer [182]

Mutation	HER2	CDH1	MET	PIK3A	P53	MSI
HER2	--	0/116 (0 %)	2/116 (1.7 %)	1/116 (0.9 %)	7/116 (6.0 %)	2/116 (1.7 %)
CDH1	--	--	2/116 (1.7 %)	1/116 (0.9 %)	10/116 (8.6 %)	0/116 (0 %)
MET	--	--	--	0/116 (0 %)	6/116 (5.2 %)	0/116 (0 %)
PIK3A	--	--	--	--	2/116 (1.7 %)	1/116 (0.9 %)
P53	--	--	--	--	--	3/116 (2.6 %)
MSI	--	--	--	--	--	--

## HER2

HER2 (encoded by ERBB2, the v-erb-b2 avian erythroblastic leukaemia viral oncogene homolog 2) is one of the four members of the human EGFR family (EGFR or HER1, HER2, HER3, and HER4) in the receptor tyrosine kinase (RTK) superfamily [15]. Unlike other HER family members, HER2 does not contain a ligand-binding site and signals through hetero-dimerization with other HER family members, primarily EGFR [19]. HER2 is expressed by normal and cancerous cells, whose gene amplification results in protein overexpression, subsequent cell proliferation, growth, and cell survival by triggering downstream signalling via the PI3K-AKT and the MAPK pathways [20, 21]. Although the prevalence, prognostic, and predictive value of HER2 is less established in GC compared to breast cancer, the importance is becoming evident as studies emerge.

HER2 has become the most important biomarker in GC. The rate of HER2 overexpression in GC has varied in the literature, ranging from 2 % [22] to as high as 91 % [23], although most studies fall between 9 and 38 % [21, 24–27]. The rate in the Trastuzumab for Gastric Cancer (ToGA) trial was 22.1 % [28]. Proposed reasons for the discrepancies include:

- *Choice of study specimen*. Some studies have used tissue microarray (TMA) [27, 29, 30], as opposed to whole slide for evaluation, and/or biopsy specimens as opposed to surgical specimens, which could be subject to sampling error due to tumour heterogeneity [31].
- *Methodology*. Different in situ hybridization (ISH) techniques provide similar results, but interpretation is easier with silver-enhanced ISH (SISH), which is an advantage when diagnosing focal amplification in very small biopsies [32–34].
- *Biological*. Relates to the intratumoural heterogeneity of HER2 alterations in GC, which has been shown to be of prognostic significance and is the main reason for discordance between IHC and FISH and between biopsies and resection specimens [35–49].
- *Location*. The HER2 overexpression/amplification rate is higher in tumours from the gastroesophageal junction than in those located in more distal parts of the stomach [40]. One study found a rate of HER2 expression in the distal stomach to be 32 % [41].
- *Type*. Intestinal-type adenocarcinomas are more commonly HER2 positive than mixed or diffuse-type neoplasms [32, 38, 42–48].
- *Differentiation*. HER2 amplification/overexpression has been associated with well to moderately differentiated tumours [22, 26, 40, 49].
- *Scoring*. In contrast to breast cancer, HER2 immunohistochemical expression in GC is more heterogeneous (focal staining) and may exhibit incomplete membrane staining. Therefore, an HER2 scoring system specific for GC has been developed with separate scoring systems for endoscopic biopsies and surgical resection specimens to ensure standardization [33].

The role of HER2 overexpression in tumourigenesis is not completely understood and its prognostic implication remains unclear [50]. HER2 overexpression seems to be an early event in gastric carcinogenesis as HER2 expression rises significantly from low-grade to high-grade dysplasia to adenocarcinoma [41]. Furthermore, the occurrence of HER2 expression in the early stage strongly suggests that there is no relationship between HER2 expression and prognosis [38]. Indeed, some reports show no difference in prognosis when compared with HER2-negative tumours [22, 25–27, 30, 49, 51–54]. However, some studies do report that HER2 amplification is associated with a poor prognosis and aggressive disease [21, 29, 55–60]. For example, a systematic review investigating the prognostic value of HER2 overexpression found that 20 studies (57 %) reported no difference in overall survival (OS), 2 (6 %) showed significantly longer OS, and 13 (37 %) significantly worse OS [61]. Interpretation of these controversial results is difficult due to lack of standardization in defining HER2 overexpression or amplification [36].

The relationships between HER2 status and other known pathologic and prognostic characteristics are more ambiguous. Some studies describe associations between HER2-positive tumours and nodal disease [30, 47], tumour size [30], depth of tumour invasion [47], and stage [47], whereas others fail to show any association [22, 56, 59]. Some reports have found no correlation between HER2 positivity and TNM stage of disease [38, 44, 46, 48]. Another study was unable to detect significant relationships between clinicopathologic factors and HER2 status with the exception that HER2-positive tumours demonstrated a lower prevalence of signet ring cell features [49]. One other study noted that 100 % of tumour samples with signet ring cell features were HER2 negative [22]. Since most studies failed to adjust for other confounders, it is difficult to interpret the reported relationship between HER2 and other histopathological variables.

HER2 expression has become the biomarker for identifying patients who are likely to show a survival benefit with trastuzumab [28]. Trastuzumab, an HER2-targeted agent, has considerable activity in HER2-positive GC but only benefits patients with HER2-overexpressing/amplified tumours [50]. The integration of targeted therapies in GC has been slower than in some other solid tumours [62], and trastuzumab is the only targeted agent approved for the treatment of advanced GC [50]. At the biomolecular level, the HER2 pathway is responsible for the repair of DNA damage (particularly, inter-strand cross-links induced by platinum analogues), so that HER2-targeted inhibition may synergize with chemotherapy and increase apoptotic stress [63]. The ToGA phase III international study assessed the efficacy in 594 patients with HER2-positive (IHC3+ or FISH+) advanced gastric or gastroesophageal junction cancer with a combination of trastuzumab + conventional chemotherapy as a treatment for GC patients. The trial demonstrated that advanced CG patients, stratified by HER2 amplification/overexpression, had longer median OS when treated with trastuzumab + chemotherapy versus chemotherapy alone (13.8 versus 11.1 months) [28]. Targeting the extracellular domain of HER2 is not the end of the story; new ways of blocking this signalling pathway are being pursued.

Lapatinib is a dual EGFR/HER2 reversible tyrosine kinase inhibitor (blocking both HER1 and HER2) that suppresses the downstream signalling involving MAPK/Erk1/2 and PI3K/Akt pathways. The efficacy of lapatinib in conjunction with paclitaxel was assessed in a randomized phase III TyTAN trial, in Asian patients with HER2-positive advanced GC. The patients who progressed on first-line therapy were randomized to lapatinib in conjunction with weekly paclitaxel versus weekly paclitaxel alone. Median OS was 11.0 months with lapatinib plus paclitaxel versus 8.9 months with paclitaxel alone (*p* = 0.10), with no significant difference in median progression free survival (PFS) (5.4 versus 4.4 months) or time to progression (5.5 versus 4.4 months). Response rate was higher with lapatinib plus paclitaxel versus paclitaxel alone (odds ratio, 3.85; *p* < .001). However, the risk of death or disease progression was significantly lower in patients with IHC 3+ tumours who were treated with lapatinib, compared with those with IHC 0/1 or IHC 2+ tumours [64]. Ado-trastuzumab emtansine (T-DM1) is an antibody-drug conjugate consisting of an antimicrotubule cytotoxic agent DM1 linked to trastuzumab. The phase II/III Gatsby trial evaluated efficacy of ado-trastuzumab emtansine in the second-line setting for the treatment of HER2-positive advanced GC. A total of 412 patients treated with first-line therapy participated in the study. The ImmunoGen, Inc., has recently disclosed that the trial did not meet its primary endpoint of OS. The trial findings have not presented yet [65].

Other HER2-directed therapies such as pertuzumab and neratinib that have demonstrated efficacy in breast cancer have not yet been evaluated in randomized clinical trials in patients with HER2-positive GC. Furthermore, dual HER2 blockade, which is an effective strategy in breast cancer, is being investigated in a phase II study using the combination of pertuzumab and trastuzumab in patients with HER2-positive metastatic GC. HER2 is a promising biomarker for targeted treatment in GC. Several clinical trials are currently exploring HER2-directed therapy in patients with GC using varied designs. The results of these future studies will be helpful to know the efficacy and tolerance of HER2-directed therapy in HER2-positive GC.

## E-cadherin

CDH1, located on chromosome 16 (q 22.1), encodes the E-cadherin transmembrane protein [66, 67]. E-cadherin is a calcium-mediated membrane molecule that plays an important role in adhesion and differentiation of gastric epithelial cells, which is a very important protective mechanism against neoplasm formation [70]. E-cadherin is one of the most important tumour suppressor genes in GC, and its inactivation is thought to contribute to tumour progression via subsequent increases in proliferation, invasion, and metastasis [15, 66, 68–72]. E-cadherin dysfunction may occur through several mechanisms, including CDH1 mutations, epigenetic silencing by promoter hypermethylation, loss of heterozygosity (LOH), transcriptional silencing by a variety of transcriptional repressors that target the CDH1 promoter, and microRNAs that regulate E-cadherin expression [67]. However, only the presence of E-cadherin structural alterations represents a poor prognostic factor [72]. E-cadherin somatic alterations exist in all clinical settings and histotypes of GC and are associated with different survival rates [72]. These alterations are, presently, non-targetable as this would require restoring E-cadherin expression by gene therapy [15]. Nevertheless, E-cadherin is a potential predictive marker of response to therapy since its impairment decreases tumour cell sensitivity to conventional and targeted therapies [72, 73]. Screening for CDH1 mutations at the time of GC diagnosis may help to predict patient prognosis and is likely to improve management of patients [71].

Multiple germline E-cadherin mutations have been reported in hereditary diffuse gastric cancer (HDGC) [74]. Analysis of families demonstrated an association between GC development and germline mutations in the E-cadherin (CDH1) gene. The CDH1 gene mutations have been scattered across the 16 exons this gene encompasses, with approximately 75 % being truncating and 25 % missense in nature [75, 76]. Moreover, there have even been large deletions of the E-cadherin gene identified in a small percentage (4 %) of HDGC families, likely involving nonallelic homologous recombination in Alu repeat regions [77]. Furthermore, 70 % of CDH1 mutation-negative HDGC probands display germline monoallelic CDH1 RNA downregulation (allelic imbalance), reinforcing the role of the CDH1 locus in this disease [78]. HDGC tumours appear when complete somatic CDH1 inactivation is acquired, leading to reduced or absent E-cadherin expression [75, 79]. This occurs through second-hit mechanisms, pursuing Knudson’s model of tumour suppressor gene inactivation [80, 81]. CDH1 promoter hypermethylation is the most frequent second-hit inactivation mechanism in HDGC primary tumours, whereas a second mutation or deletion (LOH/intragenic deletions) is less frequently identified [70, 82–84]. The cumulative risk estimate for advanced GC by 80 years of age was estimated to be 67 % for men and 83 % in women with wide confidence intervals, as these were based on 11 HDGC families [85].

A study of 42 families diagnosed with HDGC trait by having at least two members affected had an E-cadherin mutation identified in 40 % of cases. If the clinical criteria were less stringent to include only one GC occurring before 50 years of age, then more than half the cases had E-cadherin mutations [86]. When large deletions were screened in addition to point mutations and small frameshift mutations, 46 % of 160 high-risk families were found to have a germline E-cadherin gene alteration [77]. It is noteworthy that more than half to up to two thirds of HDGC families reported have proven negative for the E-cadherin gene mutation [77, 87]. Allele expression imbalance of CDH1 was noted in a subset of these families [78]; however, most of these families likely have other molecular alterations underlying their cancer predisposition that are yet to be discovered [88]. The frequency of abnormal E-cadherin expression among sporadic DGC varies and has been reported as 7 [89], 38 [66], 46 [90], and 82 % [91]. CDH1 somatic alterations were found in approximately 30 % of all patients with GC [71]. No germline mutations of this gene were detected in apparent sporadic diffuse GC cases with a mean age of 62 years in Great Britain [92]. Furthermore, a study of 25 sporadic diffuse GCs identified 1 case with a germline E-cadherin mutation and none in 14 intestinal-type GCs [93]. Molecular variables such as CDH1 alterations may be crucial to better define the survival of patients with a family history.

Abnormal E-cadherin expression may be used as a predictive factor for tumour invasiveness in gastric adenocarcinoma. One study showed a significant correlation between abnormal E-cadherin expression and tumour grade and regional lymph node involvement [66]. Another study showed that E-cadherin methylation was correlated with size of tumour, tumour stage, and nodal metastases [94]. However, one study found that E-cadherin mutation was not correlated with tumour grade or stage [95]. In keeping with most studies finding a correlation of abnormal E-cadherin expression with adverse clinicopathologic factors, tumours with CDH1 structural alterations displayed a significantly poorer survival rate than tumours negative for CDHI alterations or tumours with epigenetic CDH1 alterations [71]. For instance, one study found that patients with GC displaying CDH1 exon 8/9 deletions (structural) have a worse clinical evolution and a shorter OS [96]. Overall, abnormal E-cadherin expression favours a worse prognosis for GC patients.

## FGFR/EGFR/mTOR/MET

Important biomarkers in GC currently being investigated include the FGFR, the hepatocyte growth factor receptor (HGFR, MET), and mTOR [15].

FGFR family members (FGFR1, FGFR2, FGFR3, and FGFR4) belong to the RTK superfamily [15]. In a recent genomic survey of GC using high-resolution single-nucleotide polymorphism (SNP) arrays, FGFR2 copy number gain was found in 9 % of tumours and was more common than EGFR (8 %), HER2 (7 %), or MET (4 %) copy number gains [97]. FGFR2 has therefore attracted significant attention as a potential candidate for targeted therapy in GC [15]. The small-molecule FGFR2 inhibitor, dovitinib (TKI258), has demonstrated growth inhibitory activity in FGFR2-amplified GC cell lines and xenografts. Ongoing phase II studies will be helpful to clarify the role of dovitinib in patients with FGFR-amplified metastatic GC [97].

Phosphatidylinositol-3-kinase (PI3K)/mTOR represents one common final convergence signalling pathway originated by the activation of several RTKs. Oncogenic mutations in PIK3CA (gene encoding the alpha p110 catalytic subunit) of PI3K have been observed in GC, constitutively activating the PI3KA/mTOR pathway [15, 98]. Studies in GC have reported a mutation frequency ranging from 5 to 67 % [99–102]. In particular, EBV-positive tumours have a strong predilection for PIK3CA mutations [100]. Misregulation has been associated with increased lymph node metastases and decreased survival of GC patients [15, 103, 104]. Everolimus is an mTOR inhibitor that has shown potential benefit in advanced GC in early phase 2 trials [105, 106]. A phase 3 trial compared everolimus with placebo in 656 patients with chemotherapy refractory advanced GC [107]. Only a trivial improvement in PFS was noted (median 1.7 versus 1.4 months; *p* < 0.001). There was no significant improvement in OS (median 5.4 versus 4.3 months; *p* = 0.124).

EGFR, another member of the human tyrosine kinase receptor family, has been shown to be overexpressed by IHC in 27 % of GCs, whereas gene amplification by FISH was evident in less than 3 % in one large series [108]. Another series using FISH found an incidence of EGFR amplification to be ~5 % [109]. EGFR amplification was found to be 8 % using SNP assays [97]. EGFR overexpression has been associated with the presence of moderately or poorly differentiated histology, higher stage, and poor survival [108].

The addition of an anti-EGFR monoclonal antibody with cytotoxic chemotherapy, however, has failed to demonstrate improvement in the outcomes of patients with advanced GC. Unlike HER2 target-directed therapy, there are no established biomarkers to predict response to EGFR inhibitors. The predictive value of EGFR mutation, increased EGFR copy number, and K-ras mutation status in GC remains controversial. Two randomized clinical trials “EXPAND” and “REAL3” evaluated efficacy of an anti-EGFR monoclonal antibody (panitumumab or cetuximab) in combination with chemotherapy in patients with advanced gastric and oesophageal cancer [110, 111]. In the EXPAND trial, 904 patients with advanced GC were randomized to capecitabine and cisplatin plus cetuximab or chemotherapy alone. The median PFS of patients who received chemotherapy plus cetuximab was 4.4 months compared with 5.6 months with chemotherapy alone [110]. In the REAL3 trial, 553 patients with advanced gastric and oesophageal cancer were randomized to chemotherapy plus panitumumab or chemotherapy alone. Median OS of patients who received chemotherapy was 11.3 months compared with 8.8 months if they received chemotherapy plus panitumumab (*p* = 0.013) [111]. In addition to anti-EGFR monoclonal antibodies, orally active tyrosine kinase inhibitors (TKIs), small molecules that block the binding site of the EGFR tyrosine kinase, have been evaluated in patients with chemotherapy refractory advanced gastric and oesophageal cancer. In a phase II trial of 70 patients, erlotinib monotherapy resulted in a response rate of 9 % in patients with gastroesophageal junction cancer but none in the GC subgroup [112]. At the present time, anti-EGFR therapies do not add to conventional chemotherapy. There is a need for further investigations to identify subset of patients who will benefit from EGFR blockade.

## MET

MET (encoded by MET) belongs to the HGFR family [15]. MET is a transmembrane tyrosine kinase receptor with high affinity for hepatocyte growth factor/scatter factor (HGF/SF). Auto-phosphorylation of MET activates several signalling transduction cascades, leading to cancer cell proliferation, angiogenesis, invasion, and metastases [113].

MET amplification and/or overexpression of its protein product has long been implicated in the pathogenesis of GC, with many reports based on gene copy number, RNA expression, and/or protein expression, supporting its role as a poor prognostic factor [114–117]. Nevertheless, the prevalence of MET amplification in GC varies widely in the literature from 0 [118] to 68 % [113, 115]. This discrepancy is greatly attributed to the methodology employed to detect gene amplification/copy number gain and/or protein expression [15]. MET-positive tumours were more frequently associated with serosal invasion and other unfavourable features [118]. In all studies [97, 115–117], the GC patients with polysomic and/or amplified MET showed poorer disease-free survival and OS in comparison with the non-polysomic MET [15]. Another study has reported a significantly worse prognosis for MET-positive compared with MET-negative tumours [119]. Although MET amplification may play a central role in determining GC prognosis, future studies should focus on the possible negative predictive role for response to chemotherapy or targeted therapies [113].

Despite the fact that aberrant up-regulation of the MET/HGF pathway is associated with poor prognosis in GC, anti-MET therapies have shown limited efficacy in advanced GC. Onartuzumab is a fully humanized, mono-valent anti-MET antibody that inhibits HGF binding and receptor activation. The efficacy of onartuzumab in combination with chemotherapy (mFOLFOX6) in the first-line setting was examined for metastatic, HER2-negative gastroesophageal cancer [120]. In the MET-positive subgroup, median PFS was 5.95 months for onartuzumab and 6.8 months for placebo (HR 1.38 [0.60–3.20]). Likewise, the interim results of a phase II study of foretinib, a MET TKI, showed minimal activity in a MET-unselected patient cohort [121]. Despite early negative results, several novel MET inhibitors are now being evaluated in metastatic or unresectable GC in an attempt to identify patients who respond to MET inhibitors.

## PD-L1 expression

Programmed death-1 (PD-1) is a key immune checkpoint receptor critical for the regulation of T cell function during immunity and tolerance. The PD-1 surface receptor binds to two ligands, PD-L1 and PD-L2, which are expressed on tumour cells. PD-1-PD-L interactions control the induction and maintenance of peripheral T cell tolerance. Tumours use the PD-1 pathway to evade immune surveillance and to prevent the immune system from rejecting the tumour [122]. The frequency of programmed death ligand 1 (PD-L1) overexpression, a putative response biomarker, approaches 40 % in GC [123]. The EBV-positive sub-type of tumours has shown increased expression of PD-L1/2 [14]. Pembrolizumab is an anti-PD1 monoclonal antibody that has shown efficacy in advanced PD-1-expressing GC. In an early phase trial, 65 patients who had distinctive stromal or ≥1 % tumour nest cell PD-L1 staining were treated with pembrolizumab. The objective response rate was 22 %, and median response duration was 24 weeks. The 6-month PFS was 24 % and the 6-month OS was 69 % [124]. The results of this study have prompted expansion of immune checkpoint inhibitors in advanced GCs. Targeting the PD-1 pathway and immune checkpoint blockade appears to be a promising novel approach for the treatment of GC.

## TP53

The TP53 gene encodes a nuclear p53 protein of 393 amino acids, which acts as a potent transcription factor with a key role in the maintenance of genetic stability [125, 126]. The function of TP53 gene is usually altered through LOH, mutations, and rarely by DNA methylation [126]. TP53 mutation is one of the most prevalent genetic alterations in GC and associated with the CIN sub-type of GC [14, 126]. More than one mutation may be present in a single tumour resulting in heterogeneity of the TP53 mutational status [126].

There are conflicting results with respect to the prevalence of TP53 mutations and their relationship to histological type or tumour stage of GC. Some studies showed that mutations tend to affect mainly intestinal-type tumours, while others found that the incidence of mutation is similar in both intestinal and diffuse-type tumours, ranging between 16 and 65 % of the cases studied. The frequency of TP53 abnormalities in both early and advanced intestinal type is consistent, similar to that observed in advanced diffuse type, while in early diffuse-type TP53 mutations are uncommon [126–130].

The expression of p53 in non-tumour gastric mucosa with dysplasia was significantly higher than that in the mucosa without dysplasia. Overexpression of p53 protein was associated with the size of tumours that may help in diagnosis and prognostic prediction of GC [131]. However, the prognostic impact of p53 abnormalities on this neoplasm remains controversial. A significant association between p53 overexpression and the metastatic spread to lymph nodes or shortened survival has been described by some studies on GC but not by others [126]. At this time, p53 is not a reliable prognostic factor for GC.

## Microsatellite instability

Microsatellites are short iterations of 1–6 nucleotide long units, non-randomly distributed in both prokaryotic and eukaryotic genomes [12]. Mismatch repair (MMR) deficiency leads to a tumour phenotype known as microsatellite instability (MSI), in which cells accumulate genetic errors [15]. Several reports have shown the association of GC with MSI [132, 133]. MSI has been reported in 15 to 30 % of GC, mainly due to epigenetic silencing via hypermethylation of the MLH1 promoter [134, 135]. MSI-positive GC generally develops later in life and has a favourable prognosis when compared with MSI-negative tumours [136, 137]. The methylation of hMLH1 gene and its loss of expression increase with increasing age of the GC patient [138]. Studies have shown a strong association of MSI in GC with intestinal type, which undergoes more genomic instability in comparison to the diffuse type [12]. Moreover, MSI GC is more common in the distal part of the stomach [137, 139]. Interestingly, MSI tumours usually have an overall long-term prognosis that is favourable even in patients with advanced disease due to the fact that these tumours have a lower ability to invade serosal layers and are associated with a lower prevalence of lymph node metastases [135, 137, 139–141]. There are also higher survival rates in patients with advanced MSI GC in comparison to patients with other types of GC even with the same identical stage of the disease [142]. Information is scarce as to the prognostic value of EGFR, HER2, or VEGFA expression in the MSI subset of GC [135]. The MSI status certainly appears to be an independently positive prognostic factor, and future studies will need to determine the impact of MSI GC in the context of other co-existent molecular alterations.

## Emerging markers

### MicroRNA

MicroRNAs (miRNAs) are short fragments of noncoding RNAs comprising 18 to 24 ribonucleotides that can regulate the expression of genes by directly binding to the 3′UTR region of their target gene mRNA and impairing their translation [143, 144]. miRNAs have been found to regulate a variety of cellular processes such as cell proliferation, differentiation, invasion, migration, and epithelial-mesenchymal transition [143]. A single miRNA can regulate the expressions of thousands of genes and participate in the regulation of the whole cell cycle [145]. In detail, miRNAs negatively regulate the expression of cancer-related genes by decreasing the expression of tumour suppressor genes or enhancing the expression of oncogenes, or as modulators of cancer stem cells and metastases [146]. Accumulating evidence suggests that miRNAs play an important role in GC, but the role of specific miRNAs involved in this disease remains elusive [143].

Serum and plasma miRNAs are more stable and relatively easier to access than tissue samples [147]. The stability of tumour-associated miRNA in blood allows it to be a novel noninvasive tumour biomarker for cancer detection [148]. Circulating miRNAs must demonstrate several hallmark characteristics to be considered as reliable biomarkers: (i) stable and able to be quantified in clinical samples; (ii) present at undetectable or low levels in samples from individuals without cancer, while being expressed by cancer cells at moderate or high levels; (iii) exhibit biological functions mechanistically linked to malignant tumour progression; and (iv) provide diagnostic or prognostic information [149, 150].

The results on expression of miRNAs are inconsistent, and it is hard to select a suitable miRNA as a cancer biomarker. Furthermore, there is no consensus regarding whether plasma or serum is preferable for use as a sample, and there is a limitation to analysing the miRNA expression results of both plasma and serum [145]. Previous studies have shown that some miRNAs have been inconsistently reported when being used as a cancer biomarker [151, 152]. Possible reasons for these observed inconsistencies are the diverse experimental techniques, lack of sufficient relevant clinical data, the heterogeneous tissue samples, and poor study design [145, 153].

Numerous miRNAs have been identified as dysregulated in GC, either tissues or cell lines, many of which have also been associated with clinicopathologic features and/or survival. A number of reviews have specifically addressed the role of miRNAs in GC with comprehensive tables [154, 155]. However, to date there are no validated therapeutic trials showing miRNAs are an effective novel prognostic, predictive, or therapeutic biomarker.

### Long noncoding RNAs

Long noncoding RNAs (lncRNAs) are functional RNAs longer than 200 nucleotides [156]. According to the proximity to protein-coding genes, lncRNAs can be classified as sense, antisense, divergent or bidirectional, intronic, and intergenic [157]. As entities of transcriptional control, generally it is understood that lncRNAs may perform their functions in at least two ways: (i) as scaffoldings in ribonucleoprotein complexes, e.g. transcription or chromatin-modifying factors, acting in *cis* or in *trans* on the genome [158], and (ii) as incidental by-products of a negative type of transcriptional regulation termed “transcriptional interference” [159]. Unlike protein-coding genes, the function of these lncRNAs and their relevance to disease remain unclear [156]. Recently, a new regulatory mechanism has been identified in which crosstalk between lncRNAs and mRNA occurs by competing for shared miRNAs response elements. In this case, lncRNAs may function as competing endogenous RNAs to sponge miRNAs, thereby modulating the de-repression of miRNA targets and imposing an additional level of post-transcriptional regulation [160].

LncRNAs are still an emerging field. However, accumulating evidence has demonstrated that many lncRNAs are dysregulated in GC and closely related to tumorigenesis, metastases, and prognosis or diagnosis [156]. A total of 135 lncRNAs have been found to be aberrantly expressed in GC tissues [11, 161]. These may be potential prognostic biomarkers for GC and await future studies to further elucidate their relevance.

### Matrix metalloproteinase

The matrix metalloproteinases (MMPs) are a family of 24 zinc-dependent endopeptidases in humans that degrade components of the extracellular membrane (ECM) [162]. MMPs participate in several normal and pathological processes, and their activity is mainly modulated by the action of the tissue inhibitor of metalloproteinase (TIMP) [163]. MMPs take part in breaking down the extracellular matrix in normal physiological processes [164]. Specifically, it has been reported that both the expression of some MMP proteins and mRNA may have a large influence on GC [165, 166]. Studies regarding regulation of MMPs and TIMPs in GC have suggested that these molecules could be useful as markers of depth of invasion, metastases, and peritoneal dissemination [162]. There are some conflicting results, which are most likely related to methodological aspects and to the heterogeneity of the patient populations [167].

MMPs have been identified as up-regulated in GC, either tissues or cell lines, and have also been associated with clinicopathologic features and/or survival including MMP 3 [168], 7 [168–170], 11 [162, 168], 9 [168, 171, 172], 12 [168], 21 [168, 173], MT1 [174–176], 14 [177], 1 [162], 2 [162], and 28 [162].

MMP inhibitors, however, have shown limited clinical benefit. For example, a randomized, double-blind, placebo-controlled study evaluated efficacy of orally administered MMP, marimastat, in 369 patients with chemotherapy refractory advanced gastric and gastroesophageal cancer. A modest difference in survival was noted. The median survival was 138 days for placebo and 160 days for marimastat, with a 2-year survival of 3 and 9 %, respectively. The treatment was complicated by poor tolerability and was associated with musculoskeletal pain and inflammation [178]. Though many studies have identified the possible role of MMPs in GC, the clinical correlation is still lacking and many more studies will need to be carried out.

## Conclusions

GC is common according to global estimates of cancer and is a frequent cause of cancer-related mortality with a poor survival. At present, although the role of many genetic alterations discovered in GC seems unclear, they represent a promising tool for stratifying patients according to tumour biological behaviour and likelihood of response to systemic therapy [15]. Nevertheless, to date, except for HER2, there are no established evidence-based biomarkers predictive of tumour response to targeted agents, and the majority of patients do not yet benefit from molecularly directed therapies.

Most analyses in the literature consider a limited number of cases. As a result, despite the huge amount of data, no novel/reliable molecular marker has been introduced in the frame of secondary prevention strategies, so far [179]. It is therefore important to undertake retrospective studies in which tumour samples from patients that have undergone GC therapy are mined for single or combinations of biomarkers that can predict favourable/unfavourable response towards a certain chemotherapeutic regimen or define the use of multiple therapy regimens in GC. Independent validation of the most promising prognostic and predictive biomarkers will then be required before they can be routinely employed in clinical practice [15]. The search for a diagnostic biomarker is particularly critical for patient outcomes as early diagnosis would be instrumental in increasing survival. Future studies with identification and validation of diagnostic, prognostic, predictive, and therapeutic biomarkers will aid in the understanding of GC resulting in personalized pathway-driven targeted therapy with improved patient outcomes [180].

## Abbreviations

CIN, chromosomal instability; ECM, extracellular membrane; EGFR, human epidermal growth factor receptor family; FGFR, fibroblast growth factor receptor; GC, gastric cancer; GS, genomically stable; HDGC, hereditary diffuse gastric cancer; HGF/SF, hepatocyte growth factor/scatter factor; HGFR, MET, hepatocyte growth factor receptor; ISH, in situ hybridization; lncRNAs, long noncoding RNAs; LOH, loss of heterozygosity; miRNA, microRNA; MMPs, matrix metalloproteinases; MMR, mismatch repair; MSI, microsatellite instability; mTOR, mammalian target of rapamycin; PD-1, programmed death-1; PD-L1, programmed death ligand 1; RTK, receptor tyrosine kinase; SISH, silver-enhanced in situ hybridization; SNP, single-nucleotide polymorphism; TCGA, The Cancer Genome Atlas; TIMP, tissue inhibitor of metalloproteinase; TKIs, tyrosine kinase inhibitors; TMA, tissue microarray; ToGA, Trastuzumab for Gastric Cancer
